# Asthma is under-recognized and undermanaged in the Malaysian university population: A comparison of findings from Malaysia against Singapore from the Singapore/Malaysia cross-sectional genetic epidemiology study^☆^

**DOI:** 10.1016/j.waojou.2026.101370

**Published:** 2026-03-23

**Authors:** Qi Yi Ambrose Wong, Dingyu Cen, Jun Jie Lim, Jun Yan Ng, Jia Yi Karen Wong, Yi Ying Eliza Lim, Kashaf Nadeem, Zheng Yang Ervin Yap, Yang Yie Sio, Yee How Say, Kavita Reginald, Fook Tim Chew

**Affiliations:** a Department of Biological Sciences, Faculty of Science, National University of Singapore, Singapore; b Department of Biological Sciences, School of Medical and Life Sciences, Sunway University, Malaysia

**Keywords:** Asthma, Hypersensitivity, Surveys and questionnaires, Symptom burden, Disease management

## Abstract

**Introduction:**

Asthma is a chronic respiratory disease characterized by chest tightness, coughing, shortness of breath, and wheezing. Results from the Global Burden of Diseases Study 2021 have shown that asthma poses a notable burden on patients in Malaysia and Singapore (age-standardized prevalence rates of 2461.83 and 3352.79 per 100,000 individuals, and age-standardized disability-adjusted life year rates of 189.06 and 139.99 per 100,000 individuals, respectively). The current study aimed to compare the patterns of asthma symptoms and exacerbations between participants from Malaysia and Singapore, using data from the Singapore/Malaysia Cross-Sectional Genetic Epidemiological Study (SMCGES).

**Methods:**

The SMCGES has been ongoing since August 2005 at the National University of Singapore; Universiti Tunku Abdul Rahman; and Sunway University. Data on asthma status, symptoms, and exacerbations were obtained via a standardized and validated protocol established by the International Study of Asthma and Allergies in Childhood. Participants who indicated having ever had asthma were classified as recognized asthma cases.

**Results:**

Participants recruited in Malaysia (henceforth referred to as "Malaysian subset", n = 4028) and Singapore (henceforth referred to as "Singaporean subset", n = 11,473) were analyzed. Compared to participants from Singapore, those from Malaysia were younger (mean age ± standard deviation: 21.1 ± 4.6 vs 22.8 ± 5.7, *P* < .001) and more likely to be female (65.0% vs 58.1%, *P* < .001). The prevalence of recognized asthma was higher among subjects recruited in Singapore than in Malaysia (22.7% vs 12.0%, *P* < .001). However, asthma symptoms were significantly more prevalent among respondents from Malaysia than from Singapore. These included ever-wheezing (20.8% vs 18.9%, *P* = .01), wheezing in the past 12 months (15.8% vs 9.2%, *P* < .001), experiencing a wheezy chest after exercise (11.6% vs 4.0%, *P* < .001), and experiencing a dry nocturnal cough (13.2% vs 11.4%, *P* = .004). Participants recruited in Malaysia experienced more daytime asthma attacks (fewer than once a month: 26.1% vs 13.3%, at least once monthly: 3.3% vs 2.6%, *P* < .001) and nighttime asthma attacks (fewer than once a month: 25.6% vs 10.5%, at least once monthly: 6.1% vs 2.3%, *P* < .001) than their Singaporean counterparts. As compared to the Singaporean subset, more individuals from the Malaysian subset missed school or work at least once in the past 12 months due to wheezing or asthma (8.0% vs 2.8%, *P* < .001), visited the general practitioner or specialist for asthma (1–3 visits: 14.2% vs 9.5%, at least 4 visits: 3.4% vs 1.2%, *P* = .002), visited the emergency department for asthma (at least once: 6.0% vs 1.6%, *P* < .001), or had been admitted to the hospital for asthma (at least once: 8.1% vs 1.1%, *P* < .001).

**Conclusion:**

The prevalence of recognized asthma was higher in the Singaporean subset than in the Malaysian subset. However, there was a greater proportion of the Malaysian subset than that of Singapore that exhibited higher frequencies of wheezing, wheezing after exercising, and dry nocturnal coughing. Additionally, asthma exacerbations were more frequent in Malaysia than in Singapore. These results suggest that asthma is under-recognized and undermanaged in Malaysia.

## Introduction

Asthma is a chronic respiratory disease characterized by chest tightness, coughing, shortness of breath, and wheezing.^1^ In 2021, the Global Burden of Diseases Study (GBD) ranked asthma at 28th and 21st among Level 3 causes of global deaths and years lived with disability (YLDs).^2^^,^^3^ Although the prevalence of asthma had declined globally from 1990 to 2021, asthma continued to affect an estimated 3340 cases per 100,000 individuals, totaling approximately 260 million cases worldwide.^2^^,^^4^ Additionally, asthma resulted in an estimated 21.4 million disability-adjusted life years (DALYs), with a DALY rate of 265 per 100,000 individuals.^2^^,^^4^ For Malaysia and Singapore, age-standardized prevalence rates of 2461.83 and 3352.79 per 100,000 individuals, respectively, were obtained for asthma.^5^ Data from the GBD database showed that the age-standardized DALY rates were 189.06 and 139.99 per 100,000 individuals in Malaysia and Singapore, respectively.^5^ Hence, asthma poses a notable burden on patients in Malaysia and Singapore.

In the present exploratory investigation of individuals from university settings, we aimed to obtain an updated overview of the patterns of asthma in Malaysia and compare these results to that of Singapore using data from the Singapore/Malaysia Cross-Sectional Genetic Epidemiology Study (SMCGES). Herein, we present an updated prevalence rate of asthma, asthma symptoms, and exacerbations among Malaysians.

## Methods

### Participant recruitment

The Singapore/Malaysia Cross-Sectional Genetic Epidemiology Study (SMCGES) has been ongoing since August 2005 at the National University of Singapore; Universiti Tunku Abdul Rahman; and Sunway University. Participants were recruited via email, flyers, and poster advertisements before being screened to ensure the inclusion criteria were met and the exclusion criteria were not violated. Participants fulfilled the inclusion criteria if they were random individuals unrelated to any subject currently enrolled in the SMCGES. Conversely, individuals were excluded if they met any of the exclusion criteria: (i) had a relation to any subject who had enrolled in the SMCGES, (ii) had a known genetic disease or syndrome, (iii) had previously participated in the SMCGES, (iv) had a needle phobia, (v) had taken antihistamines within 3 days before the skin prick test (SPT), (vi) were unwilling to provide epidemiological data. Participants who were willing and able to cease antihistamine consumption were scheduled to return at least 3 days later to take part in the study.

### Data collection and skin prick test

Participants completed an investigator-administered questionnaire adapted from the International Study of Allergies and Asthma in Childhood (ISAAC) Phase Three Core and Environmental Questionnaires.^6^ Per the established and standardized protocol published by the ISAAC Steering Committee, epidemiological and symptomatologic data were collected from the study subjects.^7^

The skin prick test (SPT) was used to assess the allergy status of study subjects.^8^ Our allergen panel comprised extracts from 2 common House Dust Mite (HDM) species in Southeast Asia - *Blomia tropicalis* and *Dermatophagoides pteronyssinus*. Earlier studies have found that these HDMs are highly prevalent in Singaporean households and the local atopic population exhibits high sensitization rates to any of the 2 HDMs.^9^^,^^10^ The allergen panel included another 2 airborne allergen types – *Elaeis guineensis* pollen and *Curvularia* spp. fungal spore extracts. These aeroallergens were selected due to their high frequency of occurrence in tropical environments such as Singapore and high sensitization rates among atopic patients 11, 12, 13. Lastly, histamine and saline were included as positive and negative controls, respectively, to ensure the integrity of the SPT. During the SPT, the emergence of a skin wheal measuring at least 3 mm in diameter in response to any allergen indicated that the subject was an atopic case.

### Asthma symptoms and exacerbations

Further asthma-related data were collected using our survey questionnaire. By the ISAAC guidelines, asthma phenotypes included episodes where the chest sounded wheezy during or after exercise, and experiencing a dry cough at night which was not associated with a cold or chest infection. Among respondents who had experienced wheezing within the past 12 months, further information was obtained on the number of wheeze attacks, sleep disturbances as a result of wheezing, and wheezing severe enough to limit their speech to only 1 or 2 words between breaths.

Participants who reported ever having had asthma (ie, responded “yes” to the survey question: “Have you ever had asthma?”) were classified as recognized asthma cases (henceforth referred to as recognized asthma). Among participants with recognized asthma, data on asthma exacerbations within the past 12 months were obtained through structured survey questions evaluating: (i) frequency of daytime asthma attacks, (ii) frequency of nighttime asthma attacks, (iii) number of school or work days missed due to wheezing or asthma, (iv) number of visits to a General Practitioner or specialist for asthma, (v) number of visits to an Accident & Emergency Department for asthma, and (vi) number of hospital admissions due to wheezing or asthma. The full list of asthma-related questionnaire items and their corresponding response options is provided in Supplementary Table 1.

### Statistical analysis

The SMCGES database was analyzed using R version 4.4.0.^14^ Dichotomous (eg, yes or no) and categorical responses obtained from Malaysia using the ISAAC protocol were compared against that of Singapore using chi-squared tests. For variables involving more than 2 categorical variables (ie, 3 or more categories), chi-square trend tests (Cochran-Armitage trend tests) were used to compare the subsets. Some ordinal variables included categories corresponding to outcomes of greater severity, resulting in few (n < 5) individuals being grouped into the given category; in these cases, categories were collapsed with the preceding categories until a count of at least 5 individuals was obtained for the resultant category. Welch's two-sample t-tests were used to assess the difference between continuous variables. A threshold value of 0.05 was used to determine statistical significance.

## Results

### Participant characteristics

A total of 15,501 participants were included in the current analysis, of which 4028 were recruited in Malaysia and 11,473 were recruited in Singapore. As compared to participants recruited in Singapore, those in Malaysia were younger (mean age ± standard deviation (SD): 21.1 ± 4.6 vs 22.8 ± 5.7 years, *P* < .001), more likely to be female (65.0% vs 58.1%, *P* < .001), and mainly resided in landed properties (71.7% vs 13.1%, *P* < .001). Additionally, participants in Malaysia generally came from larger households (mean number of family members 4.9 vs 4.3, *P* < .001) and had a lower prevalence of known drug allergies (5.1% vs 11.7%, *P* < .001).

The prevalence of recognized asthma among participants in the SMCGES was 12.0% (95% confidence interval (CI): 11.0–13.1%) in Malaysia and 22.7% in Singapore (95% CI: 21.9–23.6%). Recognized asthma was significantly more prevalent among participants recruited in Singapore than in Malaysia (*P* < .001). Among local individuals born in their respective countries of recruitment, Singaporeans exhibited higher rates of recognized asthma than Malaysians (27.2% vs 12.5%, *P* < .001). Among asthma cases, the age of asthma onset among the Malaysian and Singaporean subsets was not significantly different (mean age ± SD: 5.8 ± 3.9 vs 5.5 ± 4.1, *P* = .22). Comparisons between the characteristics of the Malaysian and Singaporean subsets have been summarized in Table 1.

**Table 1:** A summary of participant demographics and recognized asthma prevalence stratified by country of collection.

Characteristic	Country of collection		*P*-value^b^
	Malaysia^a^	Singapore^a^	
**Age at collection**	21.1 ± 4.6 (4026)	22.8 ± 5.7 (11,413)	<.001
**Gender**			<.001
Female	2566 (65.0%)	6648 (58.1%)	
Male	1381 (35.0%)	4803 (41.9%)	
**Housing type**			<.001
Flats	38 (3.6%)	7297 (67.1%)	
Condominium/Private apartment	257 (24.6%)	2153 (19.8%)	
Landed property	748 (71.7%)	1426 (13.1%)	
**Income category**			<.001
Low	214 (15.3%)	2427 (21.9%)	
Low-medium	473 (33.9%)	3712 (33.4%)	
Medium-high	570 (40.9%)	2181 (19.7%)	
High	138 (9.9%)	2778 (25.0%)	
**Number of people in household**	4.9 ± 1.5 (2438)	4.3 ± 1.3 (11,264)	<.001
**History of drug allergy**			<.001
No	1800 (94.9%)	9488 (88.3%)	
Yes	96 (5.1%)	1259 (11.7%)	
**Recognized asthma**			<.001
No	3278 (88.0%)	7278 (77.3%)	
Yes	449 (12.0%)	2141 (22.7%)	
**Age of asthma onset (among asthma cases only)**	5.8 ± 3.9 (378)	5.5 ± 4.1 (1593)	0.22

a Mean ± SD (n); n (%); the sum of the number of observations may not tally with the total number of participants (or recognized asthma cases for age of asthma onset) in either Malaysia or Singapore due to missing or incomplete responses.

b Welch Two Sample *t*-test for continuous variables; Pearson's Chi-squared test for categorical variables with two categories; Chi-squared test for trend for ordinal variables with more than two categories

### Asthma symptoms were generally more prevalent in Malaysia than in Singapore

Among all participants, asthma symptoms were significantly more prevalent among respondents from Malaysia than from Singapore (see Table 2). These included ever wheezing (20.8% vs 18.9%, *P* = .01), wheezing in the past 12 months (15.8% vs 9.2%, *P* < .001), experiencing a wheezy chest after exercise (11.6% vs 4.0%, *P* < .001), and experiencing a dry nocturnal cough (13.2% vs 11.4%, *P* = .004). These trends persisted when analyses were restricted to local participants (natives) – ie, participants born in their respective country of recruitment (Supplementary Table 2).

**Table 2:** Comparisons of the proportion of asthma symptoms between participants recruited in Malaysia and Singapore. Chi-squared tests were conducted to determine the statistical significance of the difference between the proportions of affected participants from either country.

Characteristic	Malaysia N = 4,028^a^	Singapore N = 11,473^a^	*P*-value^b^
**Ever wheezing**			.01
No	2982 (79.2%)	9278 (81.1%)	
Yes	784 (20.8%)	2158 (18.9%)	
**Wheezing in the past 12 months**			<.001
No	3160 (84.2%)	8044 (90.8%)	
Yes	595 (15.8%)	811 (9.2%)	
**Wheezy chest after exercise**			<.001
No	3185 (88.4%)	8722 (96.0%)	
Yes	417 (11.6%)	365 (4.0%)	
**Dry nocturnal cough**			.004
No	3128 (86.8%)	8047 (88.6%)	
Yes	477 (13.2%)	1039 (11.4%)	

a n (%).

b Pearson's Chi-squared test; the sum of the number of observations may not tally with the total number of in either Malaysia or Singapore due to missing or incomplete responses

**Fig. 1:**
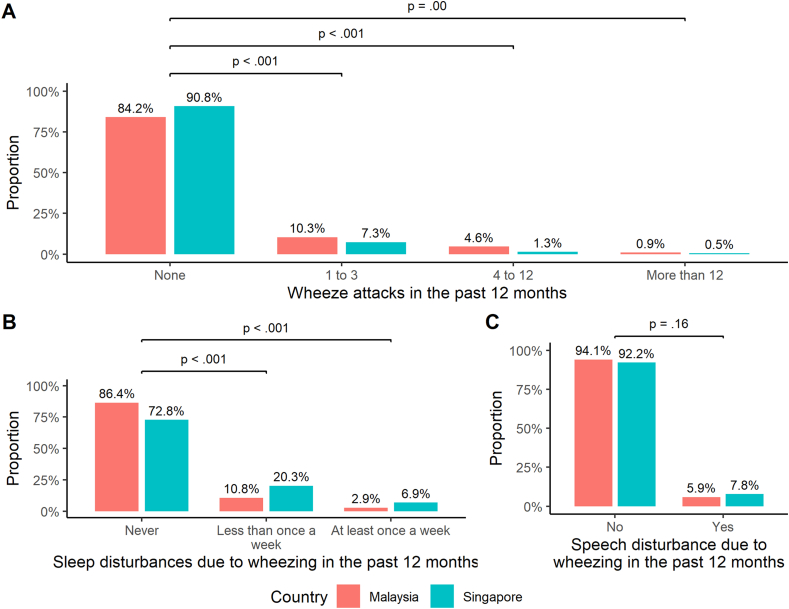
Comparisons of the proportion of wheezing outcomes in the past 12 months between participants recruited in Malaysia and Singapore, regardless of asthma status. These outcomes include (A) the number of wheezing attacks, (B) sleep disruptions due to wheezing, and (C) speech disturbances due to wheezing. Proportions indicated above the bars may not add up to 100% due to rounding. Chi-squared tests for trend and chi-squared tests were conducted to determine the statistical significance of the difference between the proportions of affected participants from either country

Among participants who had experienced wheezing episodes in the past 12 months, a significantly larger proportion of the Malaysia subset had suffered from wheeze attacks more frequently than the Singapore subset (Malaysia vs Singapore: 1 to 3 attacks – 10.3% vs 7.3%, *P* < .001; 4 to 12 attacks – 4.6% vs 1.3%, *P* < .001; more than 12 attacks – 0.9% vs 0.5%; *P* = .002; Fig. 1A). Conversely, the proportion of respondents that indicated having wheeze-induced sleep disturbances more frequently was higher in the Singapore subset than the Malaysia subset (Singapore vs Malaysia: less than once a week – 20.3% vs 10.8%, *P* < .001; at least once a week – 6.9% vs 2.9%; *P* < .001; Fig. 1B). Overall, the proportion of individuals who ever experienced sleep disruptions due to wheezing was higher in Singapore than in Malaysia (27.2% vs 13.6%, *P* < .001). Although speech disturbances due to wheezing within the past 12 months were more frequent among respondents from Singapore than Malaysia, this difference was statistically non-significant (7.8% vs 5.9%, *P* = .16; Fig. 1C). Similar trends were observed when the analysis was restricted to natives only (Supplementary Figure 1).

### Asthma exacerbations were more frequent among asthmatics in Malaysia than those from Singapore

**Fig. 2:**
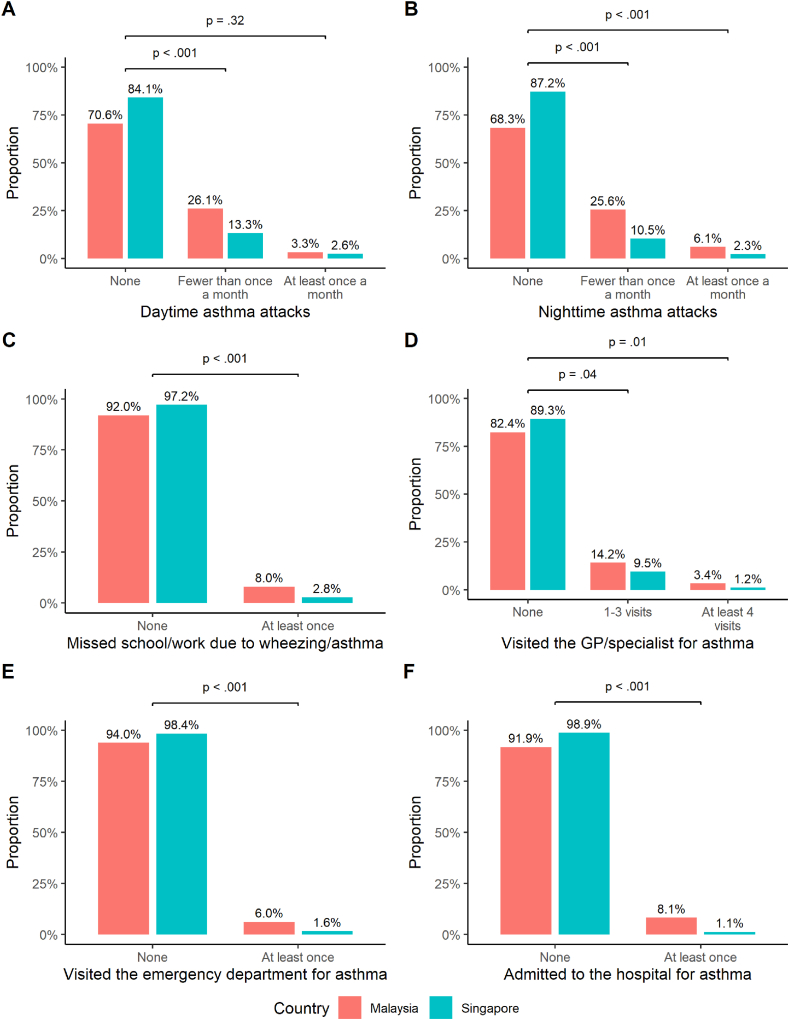
Comparisons of the proportion of asthma exacerbations in the past 12 months between participants with recognized asthma recruited in Malaysia and Singapore. These symptoms include (A) daytime asthma attacks, (B) nighttime asthma attacks, (C) missed school/work due to asthma, (D) visits to the GP or specialist due to asthma, (E) visits to the emergency department due to asthma, and (F) admissions to the hospital due to asthma. Chi-squared tests were conducted to determine the statistical significance of the difference between the proportions of affected participants from either country

Among recognized asthma cases, the trends in episode frequencies of all 6 asthma exacerbation outcomes differed significantly between the Malaysia and Singapore subsets. Participants recruited in Malaysia experienced more daytime asthma attacks (fewer than once a month: 26.1% vs 13.3%, *P* < .001; at least once monthly: 3.3% vs 2.6%, *P* < .32; Fig. 2A) and nighttime asthma attacks (fewer than once a month: 25.6% vs 10.5%, *P* < .001; at least once monthly: 6.1% vs 2.3%, *P* < .001; Fig. 2B) within the past 12 months than their Singaporean counterparts. Compared to the Singapore subset, more individuals from the Malaysian subset missed school or work at least once in the past 12 months due to wheezing or asthma (8.0% vs 2.8%, *P* < .001; Fig. 2C). Healthcare-seeking behavior associated with asthma within the past 12 months was also greater in Malaysia than in Singapore: visiting the general practitioner or specialist for asthma (1–3 visits: 14.2% vs 9.5%, *P* < .04; at least 4 visits: 3.4% vs 1.2%, *P* = .01; Fig. 2D), visiting the emergency department for asthma (at least once: 6.0% vs 1.6%, *P* < .001; Fig. 2E), and being admitted to the hospital for asthma (at least once: 8.1% vs 1.1%, *P* < .001; Fig. 2F). These trends persisted in the natives-only analysis (Supplementary Figure 2).

## Discussion

As of 2024, SMCGES has found that recognized asthma prevalence was 12.0% among participants with a mean age of 21.2 years from Malaysia and 22.7% among participants with a mean age of 22.8 years from Singapore. Our prevalence estimates for asthma were notably higher than that of the preexisting literature – in Singapore, the Singapore Mental Health Study 2016 reported an asthma prevalence rate of 11.7% among individuals aged 18 years and above, while the 2011 National Health and Morbidity Survey in Malaysia found that 6.3% of adults aged above 18 years were afflicted with asthma.^15^^,^^16^ These estimates also surpass the results from the GBD 2021 database that showed an age-standardized asthma prevalence rate of 2.60% (95% UI: 2.20–3.06) in Malaysia and 3.72% (95% UI: 3.07–4.56%) in Singapore.^17^

These variations in asthma prevalence estimates highlight possible obstacles to the identification of asthma, resulting in the under-representation of asthmatic in studies thus far. Indeed, the diagnosis of asthma has long been fraught with challenges, owing to the lack of an established gold standard for the classification of asthma, and the lack of resources available to primary care physicians.^18^ To address these issues, ongoing efforts are to maintain an updated asthma guideline, including the Global Initiative for Asthma (GINA) which has been publishing yearly reports, the latest of which was released in 2024.^1^ However, a study from 2022 found that Malaysian general practitioners (GPs) and community pharmacies were not aware of, or did not adhere to GINA guidelines in their treatment of asthma patients; most GPs did not recognize spirometry as an essential diagnostic tool and demonstrated insufficient knowledge of asthma symptoms and its treatment recommendations.^19^ Furthermore, a possible under-recognition of asthma symptoms among asthma cases could further contribute to an under-representation of asthmatics^20^, ^21^, ^22^, ^23^. We noted a dearth of current data on unrecognized asthma cases in Malaysia.

Despite asthma prevalence being lower in Malaysia than in Singapore, there was a significantly larger proportion of subjects from the Malaysian subset indicating the presence of asthma symptoms than the Singapore subset. These symptoms included ever wheezing, wheezing in the past 12 months, wheezing after exercise, and dry nocturnal coughing. Additionally, among those who had experienced wheezing in the past 12 months, a larger proportion of subjects from the Malaysia subset experienced more wheeze attacks than those from the Singapore subset.

In 2000, a cross-sectional study in Copenhagen subjected individuals with self-reported respiratory symptoms or treatments to an examination by a respiratory specialist, during which asthma was diagnosed according to their symptom history, pulmonary function test, and methacholine challenge test.^24^ Asthmatics constituted 8.42% (493/5849) of the study sample, of which 67% (331/493) were undiagnosed asthma cases; only 4.5% of respondents indicated they had doctor-diagnosed asthma.^24^ Further data from the literature showed that underdiagnosis was a constant issue across populations, examples of which included identified Danish pediatric asthma cases where as many as one-third of patients were undiagnosed, British adults where as many as 47.5% of potential asthma cases were undiagnosed, and young American adult males referred to the asthma clinic for respiratory symptoms where up to 30% of diagnosed asthma cases were previously undiagnosed^25^, ^26^, ^27^.

While there is a lack of literature on the under-recognition of asthma in Malaysia and Singapore, our current results coupled with literature from the Western world lead us to hypothesize that the true asthma prevalence in Malaysia is higher than our current estimate of 12.0%, especially given that the asthma prevalence in Singapore is estimated at 22.7% despite asthma symptoms being less prevalent among the Singaporean subset than the Malaysia subset. Further investigations focusing on the general population are imperative to elucidate the degree of asthma under-recognition among Asian countries.

Asthma exacerbations appeared to occur more frequently among individuals in Malaysia than those in Singapore – a higher proportion of participants recruited in Malaysia indicated having exacerbations at a higher frequency. The present results echo previous study findings, including that of the Asthma Insights and Reality in Asia-Pacific (AIRIAP) survey which investigated individuals who had experienced an asthma attack or symptom within the past 12 months or were using asthma medication.^28^ Not only did Malaysians exhibit asthma of greater severity (intermittent – 56.3%, mild persistent – 23.4%, moderate persistent – 12.0%, severe persistent – 8.3%) than Singaporeans (intermittent – 63.4%, mild persistent – 19.9%, moderate persistent – 12.2%, severe persistent – 4.5%), the reported asthma burden was also higher in Malaysia than Singapore with regards to hospitalization (13.2% vs 7.7%) and ER visits (12.0% vs 11.4%). Total acute care usage, comprising hospitalization, ER visits, and other unscheduled urgent care for asthma was higher in Malaysia than in Singapore (32.6% vs 27.2%).^28^ Indeed, well-controlled asthma cases consistently constitute the minority of asthmatics, with estimates showing that only 6–34% of asthma cases in Malaysia and 14% of asthmatics in Singapore were well-controlled as of 2014.^29^^,^^30^

After all, asthma control remains a multidimensional problem that involves the patient's perception of the disease, treatment compliance, and physician factors.^31^ Across the Asia-Pacific region, there has been a gross undermanagement of asthma, with the majority of asthma cases experiencing an asthma attack in the past 4 weeks (51.4%), requiring the usage of quick-relief bronchodilators (56.3%), and being limited in their physical activity (52.7%).^32^ A Malaysian study in 2021 found that a large proportion of asthmatics had limited health literacy, with 70.9% lacking an asthma action plan; moreover, 50.9% of asthmatics had uncontrolled asthma, and 87.3% of the uncontrolled cases misidentified their asthma as being controlled.^33^ A study of 6 primary healthcare providers in Malaysia found that despite possessing the appropriate resources and equipment for asthma management, these materials were not readily available in the emergency and treatment rooms.^34^ Furthermore, asthma action plans were not sufficiently implemented among asthma patients.^34^ Additionally, emergent findings show that asthma interventions are the most effective when they are adapted to the cultural perspectives, attitudes, behavior, and disease perceptions of their target population.^35^^,^^36^

A clinical diagnosis of asthma complying with GINA guidelines requires both a history of characteristic symptoms (wheeze, shortness of breath, chest tightness, or cough, that vary over time and intensity) and objective evidence of variable expiratory airflow limitation.^1^ Our study relied solely on questionnaire data to define asthma status, thus *recognized asthma* reflected a self-reported or personally acknowledged diagnosis of asthma rather than a confirmed clinical diagnosis. While this approach enabled large-scale population-level assessment between Malaysia and Singapore, our definition of asthma could be strengthened by objective lung function tests (eg, bronchodilator reversibility or spirometry) or assessment by a medical professional, particularly for subjects experiencing asthma symptoms but without a prior diagnosis. Although participants were not screened for chronic conditions that can mimic asthma, such as COPD, heart failure, chronic kidney disease, or interstitial lung disease, the prevalence of these conditions was expected to be low due to the young age of our cohort, and their impact on our findings minimal. Moreover, our current symptom data were collected according to guidelines established by the ISAAC initiative, using the ISAAC questionnaire that has been validated as for the assessment of asthma^6^^,^^37^, ^38^, ^39^. The current results provide a reliable overview of the asthma burden in Malaysia and Singapore, highlighting the under-recognition and undermanagement of asthma in Malaysia. Future research focusing on patient perceptions and management practices among asthmatics who had experienced asthma exacerbations would enhance our understanding of mismanaged asthma.

## Conclusion

Presently, data from the SMCGES has been analyzed to investigate the patterns of asthma symptoms and exacerbations. A comparison of the Malaysia and Singapore subsets showed that the prevalence of recognized asthma was higher in Singapore than in Malaysia. However, there was a greater proportion of the Malaysia subset than that of Singapore that exhibited higher frequencies of wheezing, wheezing after exercising, and dry nocturnal coughing. Additionally, asthma exacerbations were more frequent in Malaysia than in Singapore. These results suggest that asthma is under-recognized and undermanaged in Malaysia. Our findings are concordant with the existing data in the literature and reveal that the issues plaguing the management of asthma have persisted till the present time. Our findings have also highlighted a dire need for educational initiatives in the recognition of asthma symptoms among those who are unaware of the disease and asthma management among those who have been diagnosed with asthma. Not only do these interventions need to be tailored to the sociocultural factors and attitude types of their target groups, but policy shifts will also have a part to play in asthma management, especially in increasing the availability and visibility of resources such as educational materials and diagnostic equipment in primary care clinics. Ultimately, improving asthma diagnosis and management will require a multifaceted approach involving effects from multiple stakeholders.

## Abbreviations

AIRIAP, Asthma Insights and Reality in Asia-Pacific; CI, confidence interval; DALYs, disability-adjusted life years; GBD, Global Burden of Diseases Study; GINA, Global Initiative for Asthma; GPs, general practitioners; ISAAC, International Study of Asthma and Allergies in Childhood; SD, standard deviation; SMCGES, Singapore/Malaysia Cross-Sectional Genetic Epidemiology Study; SPT, skin prick test; UI, uncertainty interval; YLDs, years lived with disability;

## Authors’ consent for publication

All authors have read and consented to the publication of this manuscript.

## Availability of data and materials

All data used and included in this study are available from the corresponding author (F.T.C.).

## Author contributions

F.T.C. conceived and supervised the current research study. Q.Y.A.W. conducted the literature review, analyzed, and interpreted the data, and wrote the manuscript. Q.Y.A.W., D.C., J.J.L., J.Y.N., J.Y.K.W., Y.Y.E.L., K.N., Z.Y.E.Y., Y.H.S., K.R., and Y.Y.S assisted in recruiting study participants and data collation. All authors read and approved the final manuscript.

## Ethics approval and consent

Ethical approval for this study was granted by the National University of Singapore Institutional Review Board (reference codes: NUS-07-023, NUS-09-256, NUS-10-445, NUS-13-075, NUS-14-150, and NUS-18-036), the Scientific and Ethical Review Committee of Universiti Tunku Abdul Rahman (reference code: U/SERC/03/2016), and the Sunway University Research Ethics Committee (reference code: SUREC 2019/029). This study was performed in compliance with the Declaration of Helsinki, Good Clinical Practice, and local regulatory guidelines. Before participating, each subject was informed of this study's details via a Participant Information Sheet and provided written informed consent to participation through the signature of a Consent Form.

## Disclosure of the use of generative AI and AI-assisted technologies

Nothing to disclose.

## Funding

F.T.C. received grants from the National University of Singapore (N-154-000-038-001 (E-154-00-0017-01); C141-000-077-001 (E-141-00-0096-01)), Singapore Ministry of Education Academic Research Fund (R-154-000-191-112; R-154-000-404-112; R-154-000-553-112; R-154-000-565-112; R-154-000-630-112; R-154-000-A08-592; R-154-000-A27-597; R-154-000-A91-592; R-154-000-A95-592; R-154-000-B99-114), Biomedical Research Council (BMRC) (Singapore) (BMRC/01/1/21/18/077; BMRC/04/1/21/19/315; BMRC/APG2013/108), Singapore Immunology Network (SIgN-06-006; SIgN-08-020), National Medical Research Council (NMRC) (Singapore) (NMRC/1150/2008; OFIRG20nov-0033; MOH-001636 (OFLCG23may-0038, A-8002641-00-00)), National Research Foundation (NRF) (Singapore) (NRF-MP-2020-0004), Singapore Food Agency (SFA) (SFS_RND_SUFP_001_04; W22W3D0006; NRF-SFSRND2SIH-0001; SFS_RND_2_FS_0002), Singapore's Economic Development Board (EDB) (A-8002576-00-00), and the Agency for Science Technology and Research (A∗STAR) (Singapore) (H17/01/a0/008; and APG2013/108). This research is supported by the National Research Foundation Singapore under its Open Fund-Large Collaborative Grant (MOH-001636) (A-8002641-00-00) and administered by the Singapore Ministry of Health’s National Medical Research Council. Kavita Reginald (KR) has received funding from the T20 Research Collaboration Grant Scheme from Sunway University with Grant No.: STR-RMF-T20-005-2019. The funding agencies had no role in the study design, data collection and analysis, decision to publish, or preparation of the manuscript.

## Competing interests

F.T.C. reports grants from the National University of Singapore, Singapore Ministry of Education Academic Research Fund, Singapore Immunology Network, National Medical Research Council (NMRC) (Singapore), Biomedical Research Council (BMRC) (Singapore), National Research Foundation (NRF) (Singapore), Singapore Food Agency (SFA), Singapore’s Economic Development Board (EDB), and the Agency for Science Technology and Research (A∗STAR) (Singapore), during the conduct of the study; and consulting fees from Sime Darby Technology Centre; First Resources Ltd; Genting Plantation, Olam International, Musim Mas, and Syngenta Crop Protection, outside the submitted work. The other authors declare no other competing interests.
