# Supplementary material for: Asthma is under-recognized and undermanaged in the Malaysian university population: A comparison of findings from Malaysia against Singapore from the Singapore/Malaysia cross-sectional genetic epidemiology study
Source: World Allergy Organ J. 2026 Mar 23;19(4):101370. doi: 10.1016/j.waojou.2026.101370 (PMC13045607; doi:10.1016/j.waojou.2026.101370)
Supplement: Multimedia component 1 [file mmc1.docx]

Supplementary Table 1: Details of the asthma questionnaire used to classify asthma cases and identify asthma phenotypes.

| Question | Response options |
| --- | --- |
| Have you ever had wheezing or whistling in the chest at any time in the past? | Yes / No |
| Have you had wheezing or whistling in the chest in the last 12 months? | Yes / No |
| If "Yes", how many attacks of wheezing have you had in the last 12 months? | 1 to 3 / 4 to 12 / More than 12 / None |
| In the past 12 months, how often, on average, has your sleep been disturbed due to wheezing? | Never woken / Less than one night per week / One night per week / Twice per week / Thrice or more per week |
| In the last 12 months, has wheezing ever been severe enough to limit your speech to only one or two words at a time between breaths? | Yes / No |
| Have you ever had asthma? | Yes / No |
| In the past 12 months, has your chest sounded wheezy during or after exercise? | Yes / No |
| In the past 12 months, have you had a dry cough at night, apart from a cough associated with a cold or chest infection? | Yes / No |
| In the past 12 months, how often, on average, have you experienced asthma attacks in the daytime? | Less frequently than monthly / 1–3 times a month / 1–3 times a week / 4–6 times a week / Every day |
| In the past 12 months, how often, on average, have you experienced asthma attacks at night? | Not at all / Less frequently than monthly / 1–3 times a month / 1–3 times a week / 4–6 times a week / Every day |
| In the past 12 months, how many days (or part days) of school have you missed because of wheezing or asthma? | None / 1–5 days / 6–10 days / Once per month / More than once per month |
| In the past 12 months, how many times have you visited a General Practitioner’s clinic or Specialist's Clinic for asthma? | None / 1–3 visits / 4–12 visits / More than 12 visits |
| In the past 12 months, how many times have you visited the Accident & Emergency Department in any hospital for asthma? | None / 1–3 visits / 4–12 visits / More than 12 visits |
| In the past 12 months, how many times have you been admitted to hospital because of wheezing or asthma? | None / 1–3 times / 4–6 times / 7 or more |


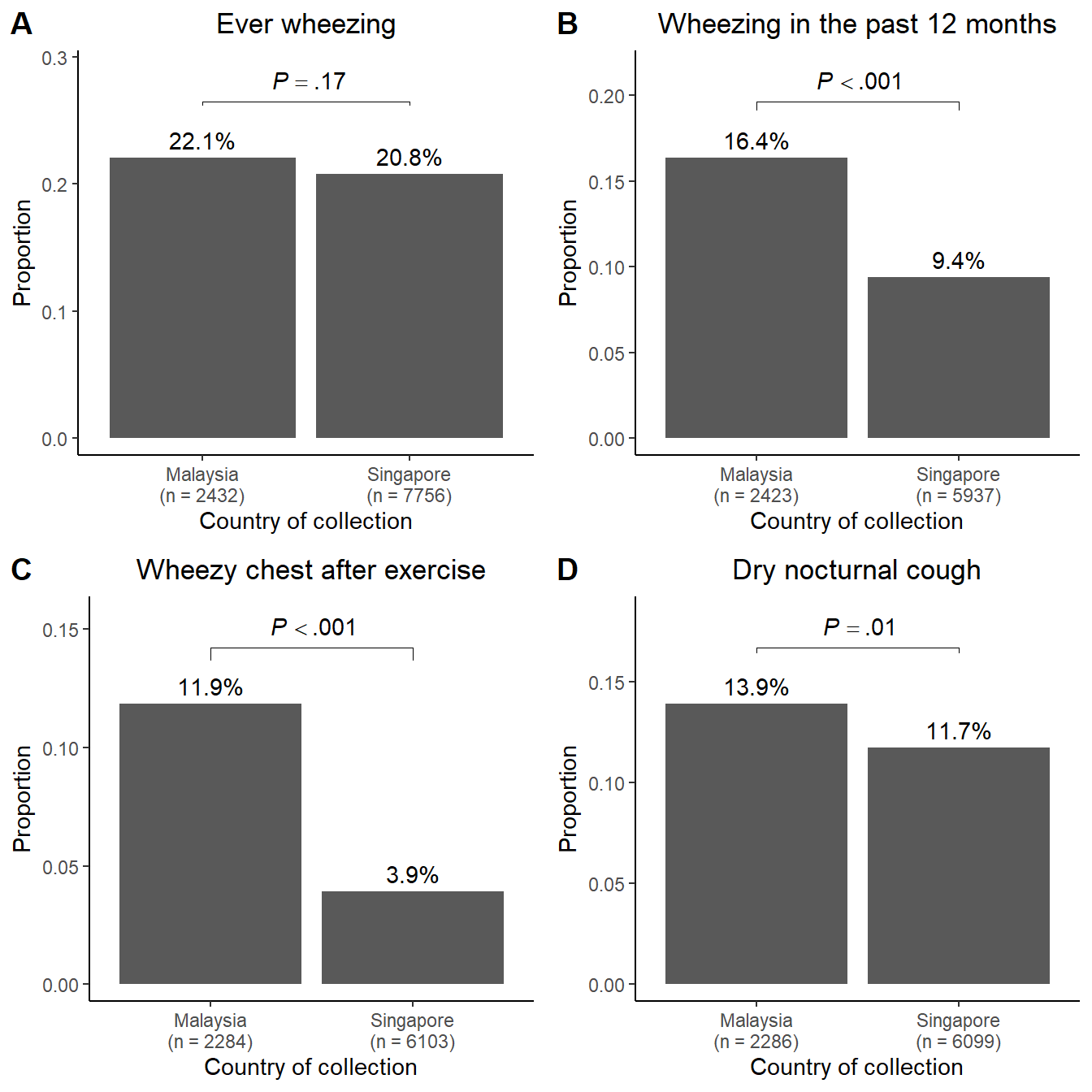
Supplementary Table 2: Proportions of ever wheezing, wheezing in the past 12 months, experiencing a wheezy chest after exercising, and experiencing a dry nocturnal cough among Malaysians born in Malaysia compared to Singaporeans born in Singapore. P-values were obtained through chi-squared tests.


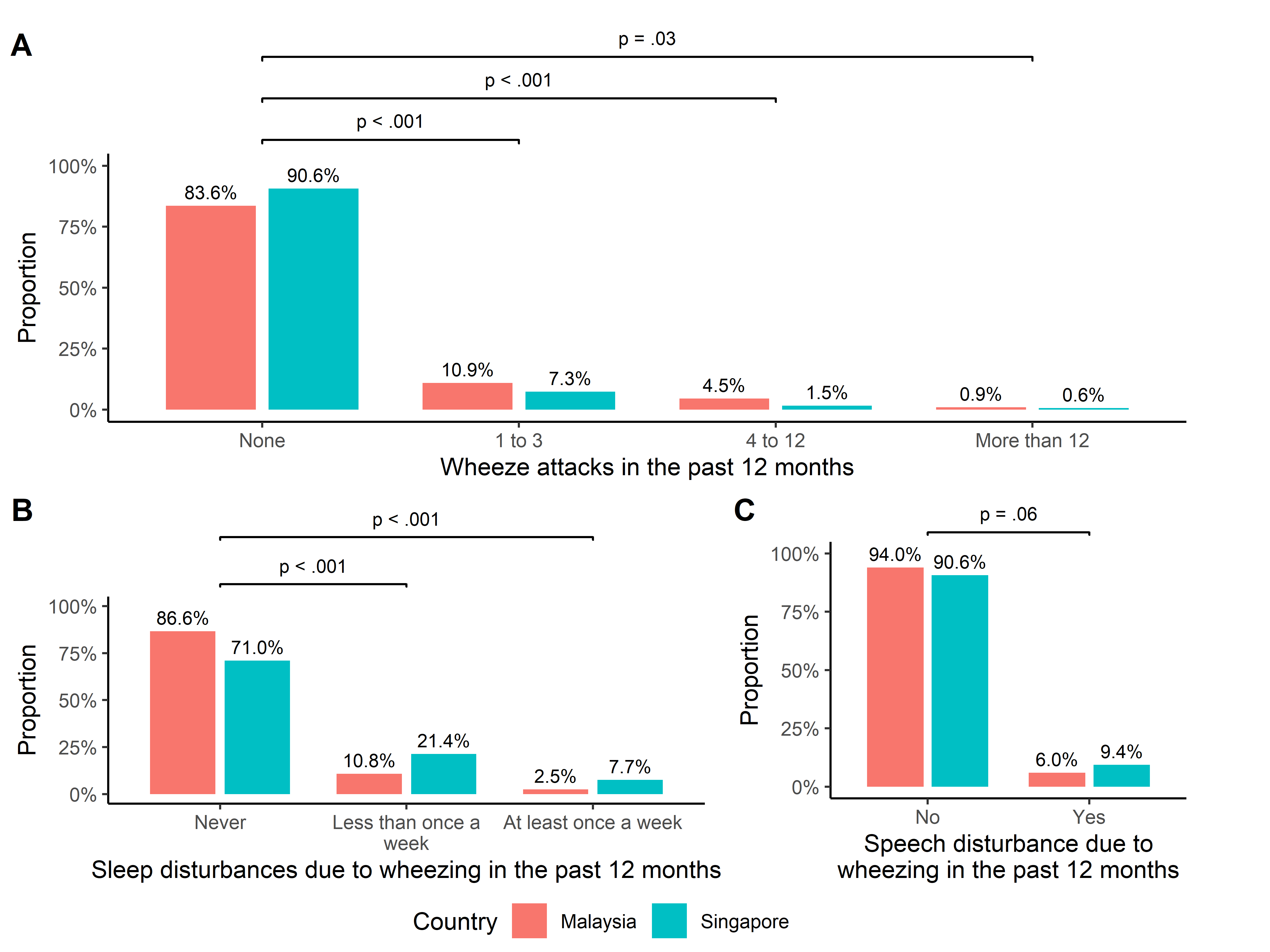


Supplementary figure 1: Trends in wheeze related outcomes among individuals who had wheezed in the past 12 months regardless of their asthma status, namely (A) number of wheeze attacks, (B) sleep disturbances due to wheezing, and (C) speech disturbances due to wheezing. Individuals included in the current analysis comprise Malaysians recruited in Malaysia and Singaporeans recruited in Singapore. Proportions indicated above the bars may not add up to 100% due to rounding. P-values were obtained through chi-squared tests for trend and chi-squared tests.





Supplementary figure 2: Comparisons of the proportion of asthma exacerbations in the past 12 months between participants with recognised asthma recruited in Malaysia and Singapore, among individuals of native ethnicity to their respective countries of collection (i.e., Malaysians recruited in Malaysia and Singaporeans recruited in Singapore). These symptoms include (A) daytime asthma attacks, (B) nighttime asthma attacks, (C) missed school/work due to asthma, (D) visits to the GP or specialist due to asthma, (E) visits to the emergency department due to asthma, and (F) admissions to the hospital due to asthma. Chi-squared tests were conducted to determine the statistical significance of the difference between the proportions of affected participants from either country.
